# Multiple endocrine neoplasia with an atypical clinical course and a *MEN1* gene variant of uncertain pathogenicity: A case report

**DOI:** 10.1097/MD.0000000000047851

**Published:** 2026-02-28

**Authors:** Syo Sato, Masahisa Arahata, Yoshihisa Kumano, Kengo Kawai

**Affiliations:** a Department of Internal Medicine, Nanto Municipal Hospital, Nanto, Japan; b Motoyoshi Clinic Affiliated With Kesennuma City Hospital, Kesennuma, Japan; c Nanto Community Medical Support Unit, Toyama University Hospital, Toyama, Japan.

**Keywords:** duodenal gastrinoma, *MEN1* gene variant, multiple endocrine neoplasia type 1, PitNET, rs2959656, SIADH

## Abstract

**Rationale:**

Multiple endocrine neoplasia type 1 (MEN1) is characterized by the coexistence of multiple endocrine tumors, most commonly due to autosomal dominant mutations in the tumor suppressor gene MEN1. Because this neoplastic disease is rare, diagnosis is often challenging. Some patients remain undiagnosed because of absent or nonspecific symptoms, while others may die before a correct diagnosis is established.

**Patient concerns:**

An 88-year-old man presenting with progressive dementia and heat stroke was admitted to our hospital. Close examinations revealed syndrome of inappropriate secretion of antidiuretic hormone and iron deficiency anemia. These conditions were attributable to a pituitary tumor and a duodenal gastrinoma, respectively, which had been identified independently by other physicians more than 6 years earlier. Genetic testing revealed an MEN1 variant of uncertain significance (rs2959656).

**Diagnoses:**

As the clinical findings fulfilled the diagnostic criteria, MEN1 was confirmed.

**Interventions and outcomes:**

His cognitive decline and behavioral symptoms improved after treatment for syndrome of inappropriate secretion of antidiuretic hormone without dementia-specific medications. Although the pituitary adenoma was initially suspected to be a prolactinoma and treated with cabergoline, insufficient tumor size reduction after 1 year of therapy led to revision of the diagnosis to a nonfunctioning pituitary neuroendocrine tumor.

**Lessons:**

This case underscores the importance of considering MEN1 even in elderly patients with atypical manifestations, emphasizes the value of integrating prior medical history into diagnosis, and suggests a potential role of rs2959656 in MEN1 pathogenesis.

## 1. Introduction

Multiple endocrine neoplasia type 1 (MEN1) is characterized by the co-occurrence of multiple endocrine tumors, most often due to autosomal dominant mutations in the tumor suppressor gene *MEN1*.^[1,2]^ Diagnosis is based on 1 of 3 criteria; presence of 2 or more primary MEN1-associated endocrine tumors (parathyroid adenoma, enteropancreatic tumor, or pituitary adenoma); occurrence of 1 MEN1-associated tumor in a first-degree relative of a patient with clinically diagnosed MEN1; identification of a germline *MEN1* mutation in an asymptomatic individual without biochemical or radiological evidence of tumorigenesis.^[1]^ Patient ages range from <10 to ≥80 years,^[1,2]^ with most cases occurring between 20 and 50 years.^[2,3]^ The prognosis is relatively poor,^[3,4]^ but may improve with presymptomatic tumor detection and targeted treatment.^[1,4]^ However, the rarity of this neoplastic disease often makes diagnosis challenging. It has been noted that some patients may remain unrecognized due to the absence of symptoms, and others may die before an appropriate diagnosis is made.^[1]^

We present a case of an older patient with MEN1 manifesting atypical symptoms, including progressive cognitive decline and behavioral and psychological symptoms of dementia, which improved after diagnosis and appropriate treatment. This case highlights the importance of thorough etiologic investigation, appropriate integration of clinical information, and consideration of factors with uncertain significance.

## 2. Case report

An 88-year-old man was brought to our emergency department with impaired consciousness and difficulty moving during hot weather in August. Heat stroke was initially suspected because of the high ambient temperature. He had been found unconscious on his electric bicycle at a location distant from his home shortly before presentation. He had not been receiving any medications at the time of admission. His past medical history included a benign pancreatic tumor, nonfunctioning pituitary adenoma, multiple gastric ulcers, a duodenal neuroendocrine tumor (NET), and mild cognitive impairment due to Alzheimer’s disease (Table 1). His mild cognitive impairment had progressed to dementia with occasional wandering. There was no family history of neoplastic diseases.

**Table 1:** Past history of the present case.

Onset	Diagnosis	Treatment and follow-up
43 years before	Pancreatic tumor	Pancreatectomy and splenectomy were performed; however, follow-up observation was not done because the tumor was identified as a benign one by histopathological examinations.
13 years before	Pituitary tumor	Close examinations (including hormonal tests) suggested that it would be a nonfunctional benign adenoma. Regular observation and follow-up were stopped 4 years later because there was no tumor progression.
7 years before	Repetitive gastric ulcer	Gastric ulcer was followed up alongside the duodenal tumor, and a proton pump inhibitor was prescribed repeatedly in the short term.
6 years before	Duodenal tumor	The tumor was identified incidentally during an upper gastrointestinal endoscopy performed for gastric ulcer. The biopsy with histopathology identified it as a Grade 1 neuroendocrine tumor. The serum gastrin concentration was found to be mildly elevated, but no additional examination was performed. He stopped visiting the hospital for regular follow-up at his own discretion.
4 years before	Mild cognitive impairment due to Alzheimer’s disease	His cognitive impairment was observed without any medication. Thereafter, it gradually developed into dementia, leading to occasional wandering.

**Figure 1.**
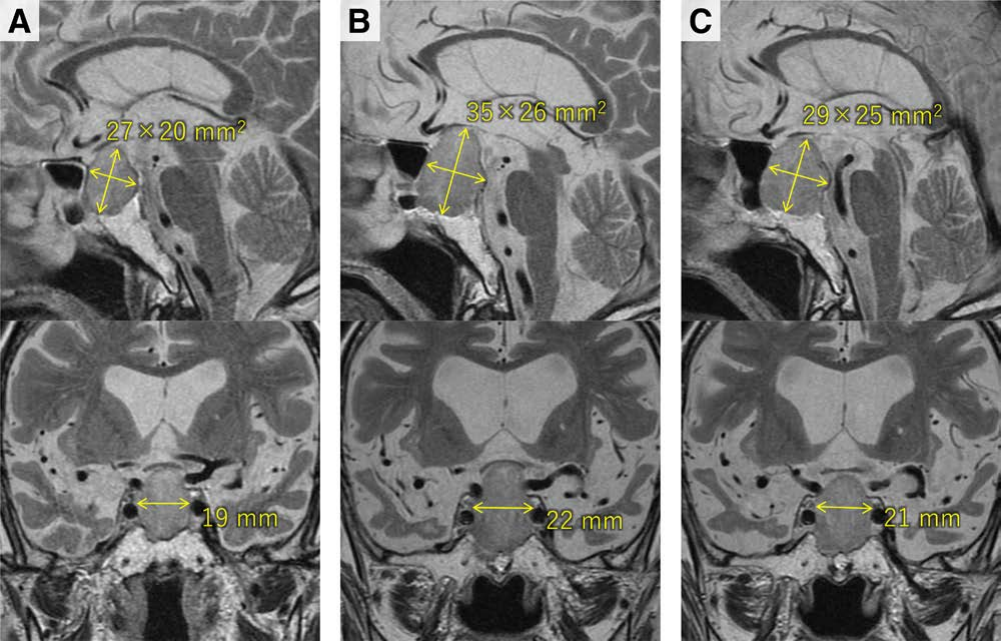
The findings of magnetic resonance imaging of the pituitary tumor. (A) The size of the pituitary tumor measured 27 × 20 × 19 mm^3^ when the last follow-up MRI was performed about 9 years before the first admission to our hospital. (B) The tumor enlarged to 35 × 26 × 22 mm^3^ at the time of the first admission. (C) The pituitary tumor shrank slightly (29 × 25 × 21 mm^3^) after 1 year of cabergoline administration at a weekly dose of 0.25 mg. MRI = magnetic resonance imaging.

On physical and neurological examination, he was disoriented (Glasgow Coma Scale was E4V4M6) but showed no other abnormal findings or edema. Laboratory findings revealed moderate anemia (hemoglobin 8.8 g/dL) and low serum concentrations of sodium (126 mEq/L), uric acid (2.6 mg/dL), and ferritin (19.0 ng/mL). Other results were within normal limits. Brain magnetic resonance imaging revealed a known pituitary tumor that had significantly increased in size compared to imaging 9 years earlier (Fig. 1). These findings suggested syndrome of inappropriate antidiuretic hormone secretion (SIADH) and iron deficiency anemia, both likely contributing to his symptoms. His consciousness improved rapidly after receiving 1 L of intravenous fluids, and he was discharged 6 days later. Thereafter, he started undergoing additional examinations (Table 2) and medical follow-up for dementia and hyponatremia. Donepezil was administered to restore his cognitive function and reduce wandering, but it was discontinued shortly afterward because of nausea. Moreover, his loss of appetite and cognitive impairment worsened, resulting in readmission 7 days after discharge.

**Table 2:** Laboratory data of the patient during the first hospitalization.

Test items	Results	Units	Reference ranges
WBC	4000	/µL	3900–9800
RBC	301	×10^4^/µL	427–570
Hemoglobin	9.4	g/dL	13.5–17.6
Hematocrit	27.3	%	39.8–51.8
Platelets	26.7	×10^4^/µL	13.1–36.2
Total protein	6.4	g/dL	6.7–8.3
AST	18	U/L	10–40
ALT	7	U/L	5–40
γGTP	11	U/L	0–70
Blood urea nitrogen	11.3	mg/dL	8.0–22.0
Creatinine	0.86	mg/dL	0.61–1.04
Uric acid	3.1	mg/dL	3.7–7.0
Serum Na	129	mEq/L	136–147
Serum K	4.1	mEq/L	3.6–5.0
Serum Cl	94	mEq/L	98–109
Serum Ca	8.6	mg/dL	8.5–10.2
Serum Fe	82	µg/dL	54–200
UIBC	241	µg/dL	104–259
Ferritin	19.0	ng/mL	39.4–340.0
ACTH	18.5	pg/mL	7.2–63.3
Cortisol	6.14	µg/dL	7.07–19.6
TSH	2.367	µIU/mL	0.61–4.23
FT4	0.73	ng/dL	0.88–1.50
Vasopressin	1.1	pg/mL	<2.8
Plasma osmolality	265	mOsm/kg	276–292
Urine osmolality	427	mOsm/kg	Not set
Urine Na	111	mEq/L	Not set
Urine Cl	105	mEq/L	Not set

The examination was carried out with blood taken following adequate infusion and recovery from dehydration. These results indicated that vasopressin secretion had inappropriately maintained in the absence of osmotic and hemodynamic stimuli and fulfilled the diagnostic criteria for SIADH.

ACTH = adrenocorticotropic hormone, ALT = alanine aminotransferase, AST = aspartate aminotransferase, Ca = calcium, Cl = chlorine, Fe = iron, FT4 = free thyroxine, K = potassium, Na = sodium, RBC = red blood cells, SIADH = syndrome of inappropriate secretion of antidiuretic hormone, TSH = thyroid stimulating hormone, UIBC = unsaturated iron binding capacity, WBC = white blood cells, γGTP = gamma-glutamyl transpeptidase.

**Figure 2.**
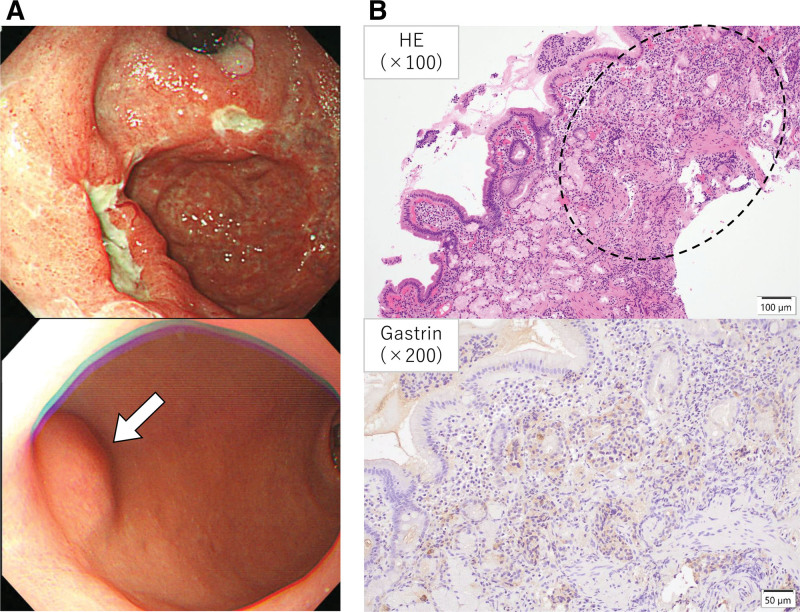
Endoscopic and histopathological findings of the gastroduodenal mucosa. (A) A photograph of upper gastrointestinal endoscopy (taken 6 years before the first admission) showed multiple gastric ulcers and a small duodenal submucosal tumor (blue arrow). Specimens were obtained from the tumor by endoscopic biopsy at that time. (B) The duodenal tumor cells tested positive for gastrin stain. On the hematoxylin–eosin stained panel, the area within the dashed ellipse indicates the tumor. HE, hematoxylin–eosin staining.

During this second hospitalization, the diagnosis of SIADH was confirmed based on laboratory findings obtained during the first hospitalization (Table 2). The administration of tolvaptan elevated his serum sodium concentration to normal levels. In addition, closer examinations were performed to clarify the etiologies of his multiple symptoms and abnormal findings. Although his pituitary adenoma had previously been identified as nonfunctioning because all serum hormone levels from the pituitary gland were within normal ranges (Table 1), reexamination revealed an elevated serum prolactin concentration (Table 3). Moreover, an additional reexamination confirmed hyperprolactinemia (prolactin 25.9 ng/mL). Thus, the pituitary adenoma was suspected to be a prolactinoma according to the diagnostic criteria in a Japanese guideline.^[5]^ Similarly, the known duodenum NET (Table 1) was suspected to be a gastrinoma because of the elevated serum gastrin concentration (Table 3) and a history of recurrent gastric ulcers. Therefore, immunohistochemical staining for gastrin was added to the duodenal mucosal specimen obtained by endoscopic biopsy 6 years earlier (Fig. 2). The result was weakly positive for gastrin (Fig. 2), leading to the final diagnosis of gastrinoma.

**Table 3:** The results of additional hormonal tests to seek endocrine tumors.

Target organs	Test items	Results	Units	Reference ranges
Pituitary gland	Prolactin	23.10	ng/mL	4.29–13.69
	GH	0.07	ng/mL	<2.47
	LH	2.4	mIU/mL	0.79–5.72
	FSH	9.5	mIU/mL	2.00–8.30
Parathyroid gland	PTH	39	pg/mL	10–65
	Calcitonin	0.57	pg/mL	<9.52
Adrenal gland	Adrenaline	36	pg/mL	<100
	Norepinephrine	334	pg/mL	100–450
	Dopamine	8	pg/mL	<20
	pVMA	11.3	ng/mL	3.3–8.6
Gastrointestinal tract	Gastrin	80.7	pmol/L	11.9–46.9

In interpreting these test results, it is important to consider that the patient was unable to remain at bed rest due to dementia and BPSD. Therefore, we focused only on the major abnormalities (which were due to the presence of tumors) and regarded minor abnormalities as artifacts.

BPSD = behavioral and psychological symptoms of dementia, FSH = follicle-stimulating hormone, GH = growth hormone, LH = uteinizing hormone, PTH = parathyroid hormone, pVMA = plasma vanillylmandelic acid.

The coexistence of a pituitary NET (PitNET) and a duodenal NET led to the diagnosis of MEN1 as per the diagnostic criteria.^[1]^ Therefore, we searched for a parathyroid tumor using neck ultrasonography and serum parathyroid hormone (PTH) levels (Table 3). However, there was no evidence to support the existence of a parathyroid tumor. As there were atypical findings, such as the absence of a parathyroid tumor and an unremarkable family history, genetic examination was performed to increase diagnostic certainty. The result showed that his *MEN1* gene had a mutation, expressed as “NM_001370259.2(MEN1):c.1621=p.Thr541=)” by the single nucleotide polymorphism database.

The duodenal NET was not resected, and an H2-receptor antagonist was administered to prevent recurrence of gastroduodenal ulcers. After beginning cabergoline for prolactinoma at a weekly dose of 0.25 mg, his serum prolactin level immediately decreased to 0.20 ng/mL. These medications were well tolerated. One year later, a slight reduction in the size of his pituitary tumor was observed on follow-up magnetic resonance imaging (Fig. 1) but the degree of tumor size reduction was insufficient to support a diagnosis of prolactinoma. Therefore, the final diagnosis of the pituitary tumor was revised to a nonfunctioning PitNET. However, his cognitive function improved by 4 points on the revised Hasegawa dementia scale (from 1 point to 5 points),^[6]^ and he had not wandered again.

## 3. Discussion

After close examinations, the patient was diagnosed with 2 NETs—a gastrinoma in the duodenum and a nonfunctioning NET in the pituitary gland. Initially, the pituitary tumor was suspected to be a prolactinoma based on the following findings: repeated measurements confirmed hyperprolactinemia; serum prolactin levels increased in parallel with tumor enlargement; and other causes of hyperprolactinemia were excluded in accordance with current guidelines.^[5,7,8]^ In addition, previous studies have reported that prolactinoma cannot be completely ruled out solely on the basis of serum prolactin concentration.^[9]^ However, the relatively low serum prolactin levels in proportion to the large tumor size and the limited radiological response to cabergoline therapy strongly suggested that the pituitary tumor was a nonfunctioning NET rather than a prolactinoma.^[7–9]^ These 2 NETs met one of the major criteria for the diagnosis of MEN1—“(1) the occurrence of 2 or more primary MEN1-associated endocrine tumors.”^[1,10]^ In addition, a single nucleotide polymorphism “NM_001370259.2(MEN1):c.1621=(p.Thr541=)” was detected during the search for *MEN1* gene variants. However, the pathological significance of this variant has not yet been determined.^[11]^

**Figure 3.**
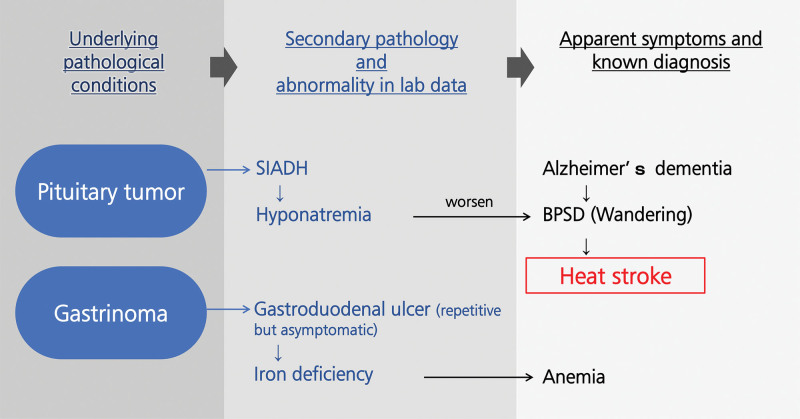
Possible etiology of the condition in this case. These findings suggest that the patient’s symptoms were largely attributable to MEN1. MEN1 = multiple endocrine neoplasia type 1.

At the time the tumors were found, we hypothesized that hyponatremia due to SIADH induced by the PitNET had worsened his dementia symptoms, such as wandering (Fig. 3). Cerebral salt wasting was not suspected because it is typically associated with intracranial disorders accompanied by hypovolemia and is rarely associated with pituitary tumors, whereas the patient was clinically euvolemic after adequate intravenous fluid administration.^[12,13]^ This pathological hypothesis (Fig. 3) was later confirmed, as he stopped wandering after serum sodium correction with tolvaptan, and his cognitive function (measured by revised Hasegawa dementia scale) improved long term after medications. This interpretation is also supported by the fact that his Alzheimer’s disease was not treated due to donepezil-induced adverse effects. His iron deficiency anemia was considered secondary to gastrinoma-induced gastrointestinal ulceration (Fig. 3). The anemia improved with iron supplementation and did not recur after administration of an H2 receptor antagonist. To our knowledge, few cases of MEN1 presenting predominantly with cognitive decline and behavioral symptoms in very elderly patients have been reported, highlighting the diagnostic challenge in this population.

In addition to delivering some critical lessons, this case highlighted important issues to be addressed. First, although his symptoms had fulfilled the diagnostic criteria for MEN1 6 years before the final diagnosis (when a duodenal NET was found), the atypical symptoms and clinical course may have delayed diagnosis. The diagnostic process was only initiated after he suffered heat stroke while wandering, an event usually considered behavioral and psychological symptoms of dementia in patients with dementia and atypical for MEN1. Another atypical feature was his advanced age, which was higher than that reported in previous cases.^[2,3]^ In addition, the absence of a parathyroid tumor was unusual, as approximately 90% of MEN1 cases present with a parathyroid.^[1,2,4]^ These characteristics should be considered alongside existing evidence when supporting the diagnosis of MEN1.

Second, his symptomatic episodes occurred sporadically over long intervals spanning more than 10 years (Table 1). Therefore, previous attending physicians may have found it difficult to identify these episodes as part of MEN1. There were, however, opportunities to suspect MEN1 if past clinical findings had been integrated. For example, the pancreatic benign tumor detected at age 45 may also have been a gastrinoma, but we could only review existing records, as the required specimen for analysis was unavailable. This case emphasizes that clinical information should be integrated appropriately at all times when treating a patient. Had the diagnosis of MEN1 been established earlier, he might have avoided heat strokes and progression of dementia.

Third, the *MEN1* gene variant, NM_001370259.2(MEN1):c.1621=(p.Thr541=) may have potential role in the etiology of MEN1, as suggested by this case. This SNP is identified as “rs2959656” by the single nucleotide polymorphism database, supported by the National Center for Biotechnology Information.^[11,14]^ Although it remains uncertain whether rs2959656 significantly contributes to MEN1 pathogenesis, previous studies have reported associations of this variant with clinically active pituitary adenomas,^[15]^ and other studies have suggested possible links to parathyroid adenomas and pancreatic insulinomas.^[16–18]^ However, its pathogenicity has not been fully established.^[11]^

Given that the global prevalence of rs2959656 is estimated at 7% to 11% regardless of race,^[14]^ MEN1 development cannot be explained solely by this variant. In our patient, we consider rs2959656 as a potential promoter of MEN1 pathogenesis, as no other MEN1 gene variants were detected. This interpretation is supported by a previous report: Nozières et al reported that 7% of patients with this SNP and a single tumor developed 2 or more tumors within 1 year.^[17]^

This case report may contribute to clarifying the pathological significance of this *MEN1* gene variant, rs2959656, which is currently of unknown significance. In conclusion, this case provides novel diagnostic insights for older patients with MEN1, highlighting the relevance of atypical presentations and SNPs with uncertain significance.

## Acknowledgments

The authors would like to thank Enago (www.enago.jp) for the initial English language review.

## Author contributions

**Conceptualization:** Masahisa Arahata.

**Data curation:** Masahisa Arahata.

**Investigation:** Syo Sato, Masahisa Arahata.

**Methodology:** Syo Sato, Masahisa Arahata, Yoshihisa Kumano, Kengo Kawai.

**Project administration:** Masahisa Arahata.

**Resources:** Masahisa Arahata, Yoshihisa Kumano.

**Supervision:** Masahisa Arahata, Kengo Kawai.

**Visualization:** Syo Sato, Masahisa Arahata.

**Writing – original draft:** Syo Sato, Masahisa Arahata.

**Writing – review & editing:** Syo Sato, Masahisa Arahata, Yoshihisa Kumano, Kengo Kawai.
